# High throughput screening of hydrolytic enzymes from termites using a natural substrate derived from sugarcane bagasse

**DOI:** 10.1186/1754-6834-4-51

**Published:** 2011-11-14

**Authors:** Severino A Lucena, Leile S Lima, Luís SA Cordeiro, Celso Sant'Anna, Reginaldo Constantino, Patricia Azambuja, Wanderley de Souza, Eloi S Garcia, Fernando A Genta

**Affiliations:** 1 Directory of Programs; National Institute of Metrology, Quality and Technology; Avenida Nossa Senhora das Graças, 50 - Xerém, Duque de Caxias, 25250-020, Brazil; 2 Zoology Department, University of Brasília, Campus Universitário Darcy Ribeiro - Instituto Central de Ciências Room AT-116, Brasília, 70910-900, Brazil; 3 Laboratory of Insect Biochemistry and Physiology, Oswaldo Cruz Institute, Avenida Brasil 4365, Leônidas Deane Building Room 207, Rio de Janeiro, 21040-360, Brazil; 4 Department of Molecular Entomology, National Institute of Science and Technology, Avenida Brigadeiro Trompowsky, Centro de Ciências da Saúde, Building D-SS room 05, Rio de Janeiro, 21941-590, Brazil

**Keywords:** bagasse, cellulase, enzyme, hemicellulase, hydrolysis, sugarcane, termite

## Abstract

**Background:**

The description of new hydrolytic enzymes is an important step in the development of techniques which use lignocellulosic materials as a starting point for fuel production. Sugarcane bagasse, which is subjected to pre-treatment, hydrolysis and fermentation for the production of ethanol in several test refineries, is the most promising source of raw material for the production of second generation renewable fuels in Brazil. One problem when screening hydrolytic activities is that the activity against commercial substrates, such as carboxymethylcellulose, does not always correspond to the activity against the natural lignocellulosic material. Besides that, the macroscopic characteristics of the raw material, such as insolubility and heterogeneity, hinder its use for high throughput screenings.

**Results:**

In this paper, we present the preparation of a colloidal suspension of particles obtained from sugarcane bagasse, with minimal chemical change in the lignocellulosic material, and demonstrate its use for high throughput assays of hydrolases using Brazilian termites as the screened organisms.

**Conclusions:**

Important differences between the use of the natural substrate and commercial cellulase substrates, such as carboxymethylcellulose or crystalline cellulose, were observed. This suggests that wood feeding termites, in contrast to litter feeding termites, might not be the best source for enzymes that degrade sugarcane biomass.

## Background

Cellulose is the most abundant biopolymer on earth, synthesized by plants at a rate of approximately 10^11 ^to 10^12 ^tons per year [1]. Lignocellulosic resources comprise a promising alternative to fossil fuels and, in the context of the global warming crisis, the need to find ways of converting these materials into usable forms of energy has become of paramount importance and may have strong socioeconomic implications, especially in developing tropical countries [2].

In Brazil, the most promising lignocellulosic resource for biofuel production is sugarcane bagasse [3]. Bagasse is a byproduct of sugar and alcohol production, obtained after the milling of the sugarcane stalk, and is currently burned by the refineries. However, research is being carried out on the hydrolysis and fermentation of sugarcane bagasse for the production of ethanol - the most important biofuel for cars in the country [4]. Techniques for pretreatment of this material by steam explosion, organosol, auto-hydrolysis, acid hydrolysis, alkaline hydrogen peroxide and alkaline extraction are being used in the so-called second generation ethanol refineries [5]. After pretreatment, the material is subjected to enzymatic treatments, and the hydrolyzed products are fermented to produce ethanol [6]. Therefore, the discovery of new enzymes with hydrolytic activity suitable for this kind of lignocellulosic material is extremely important.

One major concern with the use of commercial preparations of enzymes is that the activity detected using commercial or synthetic substrates does not necessarily reflect the activity on the natural source of the lignocellulosic material to be used. The use of a natural source of biomass for high throughput screenings is hindered by the presence of gross and insoluble particles. The utilization of a milled particle slurry for enzymatic assays with corn stover has already been proposed [7]. However, in this case the suspension was obtained after extensive physicochemical treatments of the raw material, which makes its preparation expensive and could have altered the biochemical properties of the substrate, and its reactivity to certain enzymes.

In this paper, we present a new substrate, colloidal sugar cane bagasse (CSCB), and asses it's suitability for screening hydrolases and for the characterization of enzymes from Brazilian termites; these enzymes have not yet been studied. A colloidal substrate was obtained with minimal pretreatment of the raw material, which makes the protocol cheap and suitable for high throughput screening of hydrolases.

Our group is carrying out an extensive screening of hydrolases from different organisms, ranging from bacteria and fungi to invertebrates. In this work we present the results obtained from screening invertebrate enzymes, focusing on Brazilian termites. Wide screenings of insect enzymes for biotechnological applications have been already published [8], but they are focused only on cellulase activities, using commercial substrates such as carboxymethylcellulose (CMC) or Avicel. Herein, we show a screening of termite activities with new specificities included (such as hemicellulases, xylanases and pectinases) and also show that the use of raw biomass as a substrate can reveal unexpected results. From among the termites screened, *Spinitermes nigrostomus *had the most promising activity against sugarcane bagasse. This shows that termites with non-wood feeding habits can also be an interesting source of enzymes for the digestion of lignocellulosic materials from sugarcane.

## Results and discussion

### Preparation and characterization of CSCB

After dry milling, sieving and wet milling (see Materials and methods), sugarcane bagasse is transformed into a brown and homogeneous suspension of fine particles (not shown). The suspension is not affected by autoclaving or freezing in terms of reactivity to commercial cellulolytic enzymes, and can be kept at 4°C for at least 6 months (not shown). The particles can be pelleted by centrifugation, dried by lyophilization and resuspended without loss of reactivity (not shown). However, sonication of the suspension results in a significant loss of reactivity to *Trichoderma reesei *cellulase (19 ± 2%, mean ± standard error of the mean (SEM) of three replicates).

**Figure 1:**
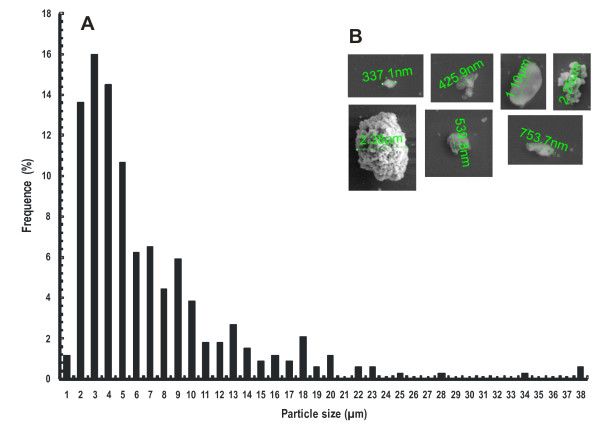
**Microscopic aspect and size of sugarcane bagasse particles present in the substrate colloidal sugar cane bagasse**. **(A) **Size distribution of particles. **(B) **Micrographs of particles.

Due to its homogeneous appearance, the suspension was named CSCB. An inspection of CSCB particles with transmission electron microscopy revealed that their size is in the micrometer range, with an average diameter of 6.0 ± 0.3 μm (mean ± SEM). The size distribution of the particles is quite broad (Figure 1A), with diameters ranging from 1 μm to 40 μm, and 56%, 36%, 7% and 1% of particles with diameters between 0 μm to 5 μm, 5 μm to 15 μm, 15 μm to 25 μm and >25 μm, respectively. An inspection of the particles with scanning electron microscopy revealed irregular shapes (Figure 1B). These features are consistent with the mild treatment and structural heterogeneity of the original material.

We were able to obtain 1.6 L of CSCB from 40 g of sugarcane bagasse, with a total sugar concentration of 2.8 ± 0.6% (w/v) (three replicates). Considering that it is possible to scale down hydrolytic assays, using 25 μL of CSCB (see below), it is possible to perform approximately 64,000 assays with a single batch of CSCB, with a low cost of US$ 0.30 per enzymatic assay. Considering the stability of the CSCB preparation and the possibility for the distribution of commercial preparations of the autoclaved suspension or of the lyophilized powder, it was deposited in the National Institute of Industrial Property of Brazil (patent number 020110064559).

### Standard enzymatic assays with commercial enzyme preparations

Before use in the screening of hydrolytic activity, the CSCB hydrolysis assay conditions were established using a commercial preparation of cellulase from *T. viride*. The release of reducing sugars from CSCB was determined with dinitrosalicylic acid, because detection with bicinchoninic acid resulted in very high background absorbances (data not shown). This could be due to the presence of proteins in CSCB, and the high reactivity of this reagent to polypeptides [9].

**Figure 2:**
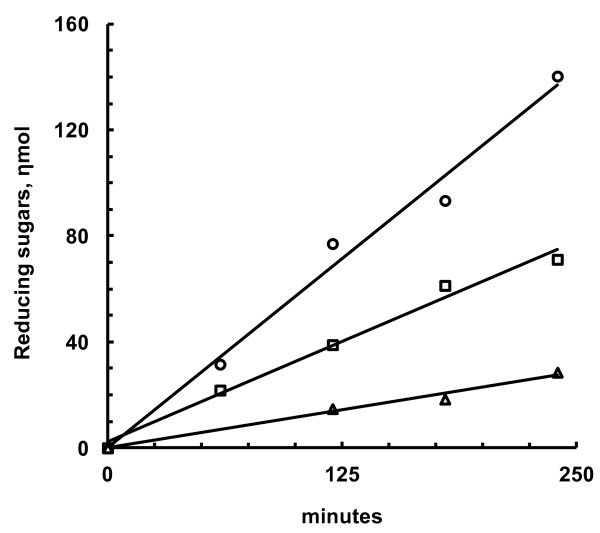
**Enzymatic assays (reducing sugars *versus *time) with a commercial preparation of *T. reesei *cellulase against various substrates**. Circles: colloidal sugar cane bagasse; squares: Avicel; triangles: carboxymethylcellulose.

The activity of *T. viride *cellulase against CSCB could be detected within short incubation times (Figure 2). Linear assays (Figure 2) were obtained even without constant mixing of reagents, and the dispersion of the suspension was stable in this condition (not shown). However, assays performed over 2 hours or longer (see below) needed constant stirring during the incubations.

The activity of *T. viride *against CSCB was similar to the activity of this enzyme against commercial substrates such as Avicel or CMC (Table 1 and Figure 2), suggesting that cellulose, which is recognized by this enzyme, is the major component of CSCB. This is consistent with the report that cellulose constitutes approximately 40% (w/w) of sugar cane bagasse [10]. Comparative analysis of activities from different animal sources against CSCB (see below) reinforces this hypothesis, even with the presence of different enzyme specificities in our commercial cellulase sample (see Table 1). Additionally, the activities measured here with CSCB and other substrates (approximately 0.2 U/mg) using the *T. viride *enzyme have the same order of magnitude as the activities described for this source of cellulase acting on filter paper (0.5 U/mg of protein to 1 U/mg protein) [11].

**Table 1:** Activities measured against different substrates and colloidal sugar cane bagasse in a commercial preparation of cellulase from *Trichoderma reesei*.

Substrate	Enzymatic activity (mU/μL)	Specific activity (mU/mg)
Laminarin	0.049 ± 0.002	290 ± 30
CMC	0.010 ± 0.001	59 ± 8
Pectin	0.0008 ± 0.0001	5 ± 1
Xylan	0.034 ± 0.002	210 ± 20
pnPβGlu	0.020 ± 0.001	120 ± 10
pnPβXyl	0.013 ± 0.001	81 ± 9
Avicel	0.015 ± 0.001	90 ± 10
CSCB	0.032 ± 0.002	190 ± 21

Protein concentration was 0.17 ± 0.01 mg/mL. Numbers are means ± standard error of the mean from five experiments. See Materials and methods for more details. CMC: carboxymethylcellulose; CSCB: colloidal sugar cane bagasse; pnPβGlu: p-nitrophenyl-β-glucoside; pNPβXyl: p-nitrophenyl-β-xyloside.

**Figure 3:**
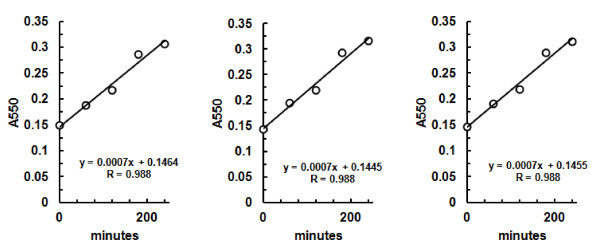
**Reproducibility of assays with CSCB**. Three different batches of CSCB were used for enzymatic assays with *T. reesei *cellulase. Linear correlation coefficients and activities are shown in each graph. CSCB: colloidal sugar cane bagasse.

The impact of using different CSCB preparations for enzyme assays was assessed in three distinct batches of this substrate with the same sample of the commercial *T. viride *cellulase. The replicates showed similar results (Figure 3), indicating that, in spite of being a biological material with a high degree of heterogeneity, CSCB could provide reproducible measurements of hydrolytic activity. This probably reflects the fact that the cellulose content of distinct batches of sugarcane bagasse does not vary very strongly [10].

The simplicity, cheap cost and miniaturization of the short term assays with CSCB indicate that this substrate could be used in the future for monitoring enzyme activity in commercial preparations, before its use on an industrial scale. This is a major concern for producers of second generation ethanol from sugarcane bagasse; the development of CSCB is part of an effort to standardize this productive chain.

### The use of CSCB for the screening of hydrolases

To test the usability of CSCB in routine screenings of hydrolytic activities in field samples, we used this substrate to monitor activities of the gut homogenate of different Brazilian termites. These animals are being subjected to intense biochemical and molecular biological research by our group as a potential source of new enzymes for use in the enzymatic hydrolysis of sugarcane bagasse. Here, we present the first results of using CSCB for enzyme screening in biological samples.

Because some termite preparations have very limited amounts of protein and activity, we used longer incubation times (up to 24 hours) for these samples, but CSCB suspension was not fully stable under these conditions. Thus, for termite samples, we performed assays in 96-deepwell plates with a small magnetic bar in each well, for stirring during incubation (see Materials and method section for details).

**Figure 4:**
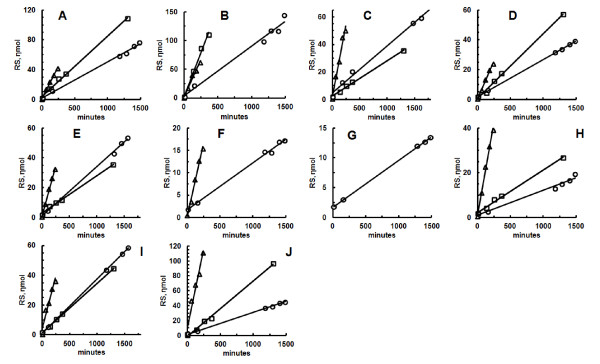
**Enzymatic assays (reducing sugars *versus *time) with carboxymethylcellulose, cellulose and colloidal sugar cane bagasse with preparations from different Brazilian termites**. **(A) ***Heterotermes tenuis*, **(B) ***Coptotermes gestroi*, **(C) ***Spinitermes nigrostomus*, **(D) ***Cornitermes silvestrii*, **(E) ***Microcerotermes strunckii*, **(F) ***Grigiotermes sp*., **(G) ***Orthognathotermes sp 2*, **(H) ***Syntermes dirus*, **(I) ***Nasutitermes jaraguae*, **(J) ***Nasutitermes bivalens*. RS: reducing sugars.

All assays of termite guts performed with CSCB showed linearity of product formation with time (Figure 4), which indicates that CSCB is suitable for the measurement of the initial rate of hydrolysis in these samples. Similar results were obtained with CMC or cellulose (Figure 4).

The hydrolytic activities against CSCB with termite gut homogenates are comparable with the activities observed when commercial substrates, such as CMC, cellulose or p-nitrophenyl-β-glucoside (pnPβGlu), are used (Table 2). Interestingly, the termite species with the highest activity against CSCB, *Spinitermes nigrostomus*, is not a typical wood feeder, being classified as a detritivore [12]. Species with the highest activities against classical cellulase substrates, that is, CMC (*Nasutitermes jaraguae *and *N. bivalens*) and cellulose (*Coptotermes gestroi *and *Heterotermes tenuis*), had poor activities against CSCB (Table 2). Therefore, we tried to understand which kind of enzyme was responsible for the high hydrolytic capacities against CSCB in *S. nigrostomus*.

**Table 2:** Activities against different substrates (including CSCB) and protein content in termites.

	Activities (mU/animal); specific activities in parentheses (mU/mg protein)								
Species	Laminarin	CMC	Pectin	Xylan	Cellulose	pNPβGlu	pNPβXyl	CSCB	Protein (mg/animal)
*H. tenuis*	0.20 ± 0.02	0.070 ± 0.004	0.11 ± 0.01	0.28 ± 0.04	1.3 ± 0.1	2.4 ± 0.1	0.36 ± 0.02	0.19 ± 0.02	0.10 ± 0.01 ^a,b^
	(2.2 ± 0.2) ^a,b^	(0.77 ± 0.07) ^a^	(1.3 ± 0.2) ^a^	(3.0 ± 0.4) ^a^	(14 ± 2) ^a^	(40 ± 2) ^d^	(6.2 ± 0.3) ^a^	(2.2 ± 0.3) ^a^	
*C. gestroi*	0.49 ± 0.05	0.14 ± 0.02	0.28 ± 0.07	0.55 ± 0.09	1.6 ± 0.2	7.3 ± 0.3	0.84 ± 0.08	0.43 ± 0.02	0.09 ± 0.01 ^a,b^
	(6.2 ± 0.6) ^a,b,c^	(1.6 ± 0.3) ^a^	(3.6 ± 0.9) ^a^	(7 ± 1) ^a,b^	(17 ± 2) ^a^	(94 ± 3) ^f^	(11 ± 1) ^a^	(4.7 ± 0.8) ^a^	
*Grigiotermes sp*.	2.7 ± 0.2	0.32 ± 0.04	0.27 ± 0.04	0.15 ± 0.04	0.022 ± 0.008	2.3 ± 0.1	2.29 ± 0.08	0.07 ± 0.01	0.14 ± 0.02 ^c^
	(19 ± 5) ^d,e^	(2.2 ± 0.8) ^a^	(1.8 ± 0.5) ^a^	(0.8 ± 0.3) ^a^	(0.20 ± 0.07) ^b^	(57 ± 5) ^e^	(58 ± 4) ^c^	(0.45 ± 0.05) ^a^	
*S. nigrostomus*	2.0 ± 0.2	1.06 ± 0.06	1.0 ± 0.2	1.4 ± 0.2	0.17 ± 0.04	2.4 ± 0.2	0.12 ± 0.02	0.930 ± 0.009	0.09 ± 0.01 ^a,b^
	(22 ± 1) ^e^	(11.7 ± 0.6) ^b^	(11 ± 2) ^b^	(16 ± 3) ^b^	(2.3 ± 0.5) ^b^	(33 ± 4) ^c,d^	(1.2 ± 0.5) ^a^	(13 ± 3) ^b^	
*M. strunckii*	0.030 ± 0.001	0.10 ± 0.01	0.10 ± 0.01	0.19 ± 0.02	0.30 ± 0.05	1.5 ± 0.2	0.17 ± 0.01	0.11 ± 0.03	0.10 ± 0.01 ^a,b^
	(0.42 ± 0.02) ^a^	(1.3 ± 0.1) ^a^	(1.23 ± 0.09) ^a^	(2.4 ± 0.3) ^a^	(2.7 ± 0.6) ^b^	(17 ± 2) ^b^	(1.9 ± 0.2) ^a^	(1.0 ± 0.4) ^a^	
*Orthognathoterme*	4.5 ± 0.4	0.13 ± 0.02	0.6 ± 0.1	0.10 ± 0.02	0	0.17 ± 0.03	0.16 ± 0.03	0.020 ± 0.005	0.26 ± 0.03 ^d^
*s sp 2*	(14 ± 1) ^c,d,e^	(0.42 ± 0.07) ^a^	(1.9 ± 0.4) ^a^	(0.32 ± 0.07) ^a^	(0) ^b^	(1.2 ± 0.2) ^a^	(1.1 ± 0.1) ^a^	(0.38 ± 0.09) ^a^	
*C. silvestrii*	2.3 ± 0.3	0.53 ± 0.09	0.57 ± 0.06	1.61 ± 0.07	0.23 ± 0.03	1.39 ± 0.01	2.67 ± 0.03	0.10 ± 0.01	0.14 ± 0.01 ^b,c^
	(19 ± 2) ^d,e^	(4.3 ± 0.8) ^a^	(4.6 ± 0.6) ^a^	(12.9 ± 0.7) ^b^	(1.5 ± 0.2) ^b^	(26 ± 1) ^b,c^	(44 ± 5) ^b^	(0.64 ± 0.04) ^a^	
*S. dirus*	5.3 ± 0.4	0.9 ± 0.3	0.59 ± 0.04	0.26 ± 0.06	0.6 ± 0.2	9 ± 1	0.7 ± 0.1	0.16 ± 0.04	0.18 ± 0.01 ^c^
	(9 ± 1) ^a,b,c,d^	(2.3 ± 0.4) ^a^	(1.0 ± 0.1) ^a^	(0.4 ± 0.1) ^a^	(4 ± 1) ^b^	(25 ± 4) ^b,c^	(2.3 ± 0.5) ^a^	(2.1 ± 0.7) ^a^	
*N. jaraguae*	0.56 ± 0.07	1.5 ± 0.1	0.80 ± 0.04	2.1 ± 0.3	0.17 ± 0.04	3.2 ± 0.1	0.4 ± 0.1	0.15 ± 0.03	0.040 ± 0.003 ^a^
	(8 ± 1) ^a,b,c^	(15 ± 1) ^b,c^	(20 ± 1) ^c^	(44 ± 7) ^c^	(3.8 ± 0.5) ^b^	(68 ± 4) ^e^	(9 ± 1) ^a^	(3.8 ± 1.1) ^a^	
*N. bivalens*	0.89 ± 0.09	1.5 ± 0.3	1.5 ± 0.1	3.15 ± 0.05	0.38 ± 0.02	1.9 ± 0.2	0.62 ± 0.02	0.140 ± 0.005	0.10 ± 0.02 ^a,b^
	(11 ± 1) ^b,c,d^	(20 ± 3) ^c^	(17 ± 2) ^c^	(37 ± 1) ^c^	(4 ± 1) ^b^	(32 ± 2) ^b,c,d^	(10.8 ± 0.2) ^a^	(1.5 ± 0.4) ^a^	

Figures are means ± standard error of the mean. See Materials and methods for details. Statistical analysis (ANOVA) was carried out with SPSS 8.0. Specific activities for each substrate were compared across species. Each column was submitted to one-way ANOVA and pairs of figures were compared using Tukey's honestly significant difference test, with significance level of 0.05. ^a,b,c,d ^In a column, groups with the same superscript letter are not significantly different. CMC: carboxymethylcellulose; CSCB: colloidal sugar cane bagasse; pnPβGlu: p-nitrophenyl-β-glucoside; pNPβXyl: p-nitrophenyl-β-xyloside.

The ratio between the activities against cellulose and CMC has been used as an indicator of the presence of enzymes with binding domains that are able to recognize crystalline or insoluble forms of cellulose, such as cellobiohydrolases or specific (in many cases processive) endo-β-1,4-glucanases [13]. Strikingly, *S. nigrostomus*, the species with the highest activity against CSCB, had a low cellulose to CMC hydrolysis ratio when compared to other termites (Table 3). Some termites (*H. tenuis *and *C. gestroi*) presented cellulose to CMC hydrolysis ratios that were higher than the commercial *T. viride *cellulase (Table 3) as well as high CSCB to CMC hydrolysis ratios, but they had the smallest CSCB to cellulose hydrolysis ratios. Additionally, *S. nigrostomus *had the highest CSCB to cellulose hydrolysis ratio (Table 3). Taken together, these facts suggest that components other than cellulases could be responsible for the hydrolysis of CSCB in these digestive systems.

**Table 3:** Ratio between activities against selected substrates in termites and *T. reesei*. Ratios and deviations were calculated using data from Tables 1 and 2.

Species	Cellulose/CMC	CSCB/CMC	CSCB/Cellulose
*Heterotermes tenuis*	19 ± 3 ^c^	3.1 ± 0.8 ^b,c,d^	0.16 ± 0.03 ^a^
*Coptotermes gestroi*	12 ± 3 ^b,c^	3 ± 1 ^c,d^	0.28 ± 0.01 ^a^
*Grigiotermes *sp.	0.14 ± 0.05 ^a^	0.28 ± 0.06 ^a,b^	1.8 ± 0.4 ^a^
*Spinitermes nigrostomus*	0.19 ± 0.03 ^a^	1.1 ± 0.3 ^a,b,c^	6 ± 1 ^b^
*Microcerotermes strunckii*	2.0 ± 0.3 ^a^	0.7 ± 0.2 ^a,b^	0.37 ± 0.07 ^a^
*Orthognathotermes sp 2*	0 ^a^	1.2 ± 0.6 ^a,b,c^	-
*Cornitermes silvestrii*	0.35 ± 0.02 ^a^	0.17 ± 0.03 ^a^	0.4 ± 0.1 ^a^
*Syntermes dirus*	6 ± 2 ^a,b^	2.0 ± 0.6 ^a,b,c,d^	1.3 ± 0.6 ^a^
*Nasutitermes jaraguae*	0.23 ± 0.03 ^a^	0.3 ± 0.1 ^a,b^	1.2 ± 0.4 ^a^
*Nasutitermes bivalens*	0.23 ± 0.07 ^a^	0.08 ± 0.02 ^a^	0.36 ± 0.02 ^a^
*Trichoderma reesei*	1.98 ± 0.08 ^a^	4.25 ± 0.08 ^d^	2.14 ± 0.05 ^a^

Statistical analysis (ANOVA) was carried out with SPSS 8.0. Ratios between activities were compared across species. Each column was submitted to one-way ANOVA and pairs of figures were compared using Tukey's honestly significant difference test, with significance level of 0.05. ^a,b,c,d ^In a column, groups with the same superscript letter are not significantly different. CMC: carboxymethylcellulose; CSCB: colloidal sugar cane bagasse.

To carry out a more detailed study, we compared the enzymatic activities of termites using CSCB and the commercial substrates. Specific activities against p-nitrophenyl-β-xyloside did not show positive correlation with activities against CSCB (r = -0.35). Other activities showed low positive correlations with CSCB hydrolysis, but these were not statistically significant (laminarin, r = 0.33; CMC, r = 0.34; pectin, r = 0.34; xylan, r = 0.21; pnPβGlu, r = 0.22; cellulose, r = 0.13). These results suggest that the concerted action of several enzymes could be determinant of the efficient hydrolysis of CSCB, rather than only one enzyme specificity. Nevertheless, we cannot discard the possible action of other hemicellulases, or synergistic effects between enzymes, to explain the high activity of *S. nigrostomus *against CSCB. There is an interesting parallel between the presence of litter from grasses in the diet of this insect and the hydrolysis against CSCB; this suggests that, for the hydrolysis of sugarcane material, the enzymes from wood feeding termites might not be the most interesting.

There has been an extensive increase in studies concerning termite hydrolytic enzymes and digestive physiology [14,15]. The isolation, characterization, cloning and expression of hydrolytic enzymes from these insects have already been reported [16,17].The gut microbiota of some termite species has already been the subject of detailed transcriptomic and metagenomic studies [18-20], and several genes with biotechnological potential have been sequenced. In spite of this, the digestion physiology in termites is a poorly studied subject, considering that only a few cases from a group of 2,600 known species [21] have been subjected to any kind of biochemical study. Our findings show that termites are probably an important source for the discovery of new enzymes.

More importantly, our data clearly shows that screening with a natural substrate can result in enzymatic profiles quite different from those obtained with commercial cellulase substrates, such as CMC and crystalline cellulose. The choice of substrate that best represents the material to be used in the biodegradation processes can be of major importance for decision making in this field of biotechnological research.

## Conclusions

We have shown that raw sugarcane bagasse can be transformed into a colloidal suspension of particles suitable for high throughput enzymatic assays. We also found that screening of termite gut homogenates using a natural substrate identified a species, *S. nigrostomus*, with high activity against sugarcane bagasse. This highlights the important differences in screening using natural substrates compared with commercial cellulase substrates.

## Methods

### Termites

Adult workers were collected in the states of Rio de Janeiro (RJ) and Minas Gerais (MG), Brazil. *Grigiotermes *sp. and *Cornitermes silvestrii *were collected in the Parque Estadual do Sumidouro (latitude -19.5615°, longitude - 43.9588°) in the municipality of Lagoa Santa (MG). *Orthognathotermes sp 2 *and *S.nigrostomus *were collected near the city of Lagoa Santa, and *H. tenuis *was collected inside a sugarcane silo also in this region (MG, latitude -19.5804°, longitude - 43.9498°). *N. bivalens *was collected in the municipality of Laje do Muriaé (RJ, latitude - 21.2095°, longitude - 42.1281°). *N. jaraguae*, *Microcerotermes strunckii *and *C. gestroi *were collected in the FIOCRUZ campus (RJ, latitude - 22.8771°, longitude - 43.2432°). *Syntermes dirus *was collected in the campus of the Universidade Federal do Rio de Janeiro (RJ, latitude - 22.8432°, longitude - 43.2349°). Insects were identified in the field under a stereomicroscope, using Constantino's illustrated key [12], and 50 to 100 individuals from each species (including all developmental stages) were sent in ethanol 70% (v/v) to the Isoptera Collection of the Universidade de Brasília (RC) for final identification and preservation.

### Preparation of samples

Right after capture, insects were immobilized on ice and dissected in cold 0.25 M sodium chloride. The entire gut was pulled apart and, after removal of the Malpighian tubes and fat body, tissues were rinsed with saline and frozen in liquid nitrogen. Five sets of forty animals were dissected for all but three species: *N. jaraguae *(three sets of forty individuals), *S. dirus *(five sets of thirteen individuals) and *Orthognathotermes sp 2 *(five sets of thirteen individuals). After transportation to the laboratory, samples were kept at -20°C.

Termite guts were homogenized in 1 mL (except for *S. dirus*, homogenized in 2 mL) of double distilled water using a Potter-Elvehjem homogenizer with 10 strokes, and used directly on enzymatic assays.

### Preparation of CSCB

Sugar cane bagasse was provided by an ethanol producer from the city of Campos dos Goytacazes (RJ). Forty grams of the bagasse were macerated in 200 mL of liquid nitrogen in a mortar. The resulting material was ground in an analytical mill (model Q298A21, Quimis, Brazil, Diadema) with two periods of 1 minute, after which it was macerated again as described above and ground for a further minute. The material was then passed sequentially through three sieves (19 mesh/cm, 26 mesh/cm, US Standard and a circular mesh with 0.8 mm diameter holes, 121 holes/cm^2^). Each fraction, with the different particle sizes, was separately milled in a ceramic ball mill (model 298-1, Quimis) with 400 mL of double distilled water. The resulting colloidal suspensions were then autoclaved and combined in proportion to the raw material in a laminar flow cabinet and, when needed, used directly in the enzymatic assays. All steps were monitored by quantifying total carbohydrates with the phenol-sulfuric method [22].

### Protein measurements and enzymatic assays

Protein was determined with Coomassie Blue G using ovalbumin as a standard [23]. The activity against soluble polysaccharides and CSCB was determined by measuring the release of reducing groups with dinitrosalicylic acid [24] with 0.25% (w/v) laminarin (from *Laminaria digitata*, Sigma Chemical Company, Saint Louis, MO, USA; catalogue number L9634), CMC, pectin (from apples, Fluka; catalogue number 76282) and xylan (from birch wood, Fluka; catalogue number 95588) or 0.21% (w/v) CSCB. β-glucosidase and β-xylosidase activity were determined by measuring the release of 4-nitrophenolate [25] with 5 mM 4-nitrophenyl-β-glucoside and 4-nitrophenyl-β-xyloside, respectively. Cellulase activity was determined by measuring the release of reducing groups with bicinchoninic acid [26] with 0.25% (w/v) crystalline cellulose (Sigma; catalogue number S5504). The CSCB and cellulose suspensions were maintained under shaking throughout the assay. All chemicals and substrates (except CSCB) and *T. reesei *crude cellulase (catalogue number C7377) were acquired from Sigma Chemical Company.

Unless otherwise specified, all substrates were assayed in 50 mM citrate-sodium phosphate, pH 6.0 at 30°C under conditions such that activity was proportional to protein concentration and time of incubation. Controls without enzyme or without substrate were included. One unit of enzyme (U) was defined as the amount that hydrolyzes 1 μmol of bonds per minute.

### Analysis of particles in CSCB by transmission and scanning electron microscopy

For transmission electron microscopy, a CSCB suspension obtained as described above was deposited in a copper grid covered with Formvar/Carbon film and analyzed in a FEI Tecnai Spirit 120 Microscope (FEI, Hillsboro, Oregon, USA), operating at 80 kV. Particle diameters were measured using the program Megaview 3 Imagen System Analysis 3.1 (FEI, Hillsboro, Oregon, USA).

For scanning electron microscopy, samples were deposited onto a glass slide previously treated with poly-L-lysine. The samples were then dehydrated using increasing concentrations of ethanol, dried with the Supercrytical Dryer Leica EM SCD 500 (LEICA, Wetzlar, Germany) and covered with 2 nm of gold in the Sputtering LEICA EM CPD 030 (LEICA, Wetzlar, Germany). Samples were then observed using the FEI Novananolab 600 (FEI, Hillsboro, Oregon, USA) operating at 2 kV.

## Abbreviations

CMC: carboxymethylcellulose; CSCB: colloidal sugar cane bagasse; MG: Minas Gerais; pNPβGlu: p-nitrophenyl-β-glucoside; RJ: Rio de Janeiro; SEM: standard error of the mean.

## Competing interests

The CSCB preparation was deposited in the National Institute of Industrial Property of Brazil (INMETRO; patent number 020110064559). This work was partially funded by Petrobas (Centro de Pesquisa e Desenvolvimento Leopoldo A Miguez de Mello; CENPES) and the National Institute of Metrology, Quality and Technology, which hold the patent rights together with SAL, WS, ESG and FAG.

## Authors' contributions

SAL, LSL and FAG carried out the CSCB preparations, collection and preparation of termite samples and biochemical assays. LSAC and CS carried out the transmission and scanning electron microscopy experiments and data analysis. FAG and SAL analyzed the biochemical data. RC identified the termites and participated in the discussion of termite ecology and physiology. FAG, SAL, PA, WS and ESG conceived the study and designed the experiments. FAG drafted the manuscript, with the help of SAL and CS. All authors read and approved the final manuscript.
